# Germline genetic testing in prostate cancer – further enrichment in variant histologies?

**DOI:** 10.18632/oncoscience.403

**Published:** 2018-04-29

**Authors:** Mark C. Markowski, Emmanuel S. Antonarakis

**Affiliations:** Departments of Oncology and Urology, Johns Hopkins Sidney Kimmel Cancer Center, Baltimore, MD 21287, USA

The genetics and heritability of prostate cancer have been well studied and continue to be elucidated. Recently, germline mutations in homologous recombination (HR) DNA repair genes have been observed in a significant proportion of men with prostate cancer. Based on the pioneering work in breast and ovarian cancers, pharmacological targeting of the poly-ADP ribose polymerase (PARP) enzyme has been shown to induce synthetic lethality in HR-deficient tumors. The discovery and characterization of germline HR mutations in prostate cancer patients may increase therapeutic options and lead to improved clinical outcomes.

HR DNA repair gene mutations have been identified in both primary hormone-sensitive [1] and metastatic castration-resistant prostate cancer (mCRPC) [2]. With respect to germline mutations, a seminal study by *Pritchard et al* found that the overall prevalence of pathogenic inherited DNA repair gene alterations in metastatic prostate cancers (11.8%) was significantly higher than in men with localized prostate cancer (4.6%) or in healthy men from the general population at large (2.7%) [3]. Specifically, mutations in seven genes (*BRCA2, ATM, BRCA1, CHEK2, PALB2, GEN1, RAD51D*) were significantly enriched in patients with metastatic prostate cancer. These prevalence estimates have been corroborated by other studies, as summarized in (Table 1). These findings suggested that a subset of men may be more likely to develop metastatic, and potentially more aggressive, prostate cancer (*i.e.* those with germline mutations in DNA repair genes).

**Table 1:** Overview of Prevalence (#patients/total patients) of Pathogenic Germline Mutations in Homologous Recombination DNA Repair Genes in Metastatic Prostate Cancer

	Pritchard et al (3)	Annala et al (4)	Antonarakis et al (6)	Isaacsson et al (8)	Total
Gene					
*BRCA2*	5.3% (37/692)	5.0% (16/319)	2.9% (5/172)	6.0% (9/150)	**5.0%**(67/1333)
*ATM*	1.6% (11/692)	0.3% (1/319)	1.7% (3/172)	2.0% (3/150)	**1.4%**(18/1333)
*CHEK2*	1.9% (10/534)	0% (0/319)	0.6% (1/172)	2.0% (3/150)	**1.2%**(14/1175)
*BRCA1*	0.9% (6/692)	0.3% (1/319)	0.6% (1/172)	1.3% (2/150)	**0.8%**(10/1333)
*PALB2*	0.4% (3/692)	0.6% (2/319)	0% (0/172)	0.7% (1/150)	**0.5%**(6/1333)
**All 5 genes**	10.1% (70/692)	6.3% (20/319)	5.8% (10/172)	12.0% (18/150)	**8.9%**(118/1333)

Although androgen deprivation therapy (ADT) is the backbone of treatment for metastatic disease, there are conflicting studies describing the efficacy of standard ADT in patients with germline deficiencies in HR DNA repair pathways. *Annala et al* found the overall prevalence of germline DNA repair mutations in their metastatic prostate cancer cohort to be 7.5% (22/319) [4]. Patients harboring germline HR DNA repair mutations had significantly shorter time from ADT initiation to the development of castration resistance (11.8 vs. 19.0 months), and progression-free survival (PFS) on first- line novel AR-targeted therapy was shorter (3.3 vs. 6.2 months) compared to non-mutated patients. In that study, no difference in PFS with chemotherapy was observed. *Mateo et al* recently performed a similar analysis on a subset of patients analyzed in the *Pritchard et al* study [5]. The treatment patterns of 60 germline-mutated patients were retrospectively evaluated. Although time to castration resistance was not studied, PFS on novel AR-targeted therapy was not significantly different based on germline mutation status. Similar to the *Annala et al* study, PFS on chemotherapy was equivalent between cohorts. Interestingly, our group has observed improved PFS (hazard ratio 0.52, p=0.044, and overall survival (hazard ratio 0.34, p=0.048) on multivariable analyses in mCRPC patients with germline *BRCA1/2* or *ATM* mutations compared to non-mutated patients [6]. These findings suggest that the presence of HR DNA repair mutations may confer a more favorable response to AR- targeted agents, although a clear consensus has not been reached. Taken together, the role of standard-of-care therapies in HR DNA repair-deficient prostate cancer needs further clarification.

However, the presence of germline HR DNA repair mutations may be a therapeutic liability when treating these patients with a PARP inhibitor, irrespective of prior response to ADT. In the previously mentioned retrospective study by *Mateo et al*, use of a PARP inhibitor or platinum-based chemotherapy resulted in an overall survival hazard ratio of 0.59 (95% CI 0.28-1.25; p=0.17), a strong trend towards an improved outcome. Moreover, a single-arm Phase 2 study (TOPARP-A) using olaparib in men with mCRPC led to an enriched response rate to PARP inhibitor therapy in those patients with deficient HR DNA repair [7]. The presence of a DNA repair defect (either somatic or germline) correlated with improved response rates (88% vs. 6%) as well as improved progression-free (9.8 vs. 2.7 months) and overall (13.8 vs. 7.5 months) survival in that trial. Notably, all 7 patients with *BRCA2* alterations achieved a 50% PSA response, and 3 of 5 patients with *ATM* mutations had a clinical response to PARP inhibition. Based on the results from the TOPARP-A trial, further study of PARP inhibitors in prostate cancer is warranted.

One of the challenges in conducting clinical trials specifically for patients with germline-mutated DNA repair genes is the large number of patients that need to be screened to identify eligible men. To this end, a recent retrospective study from our group[8] may have uncovered a unique association between germline HR DNA repair mutations and the presence of intraductal or ductal pathology, histological variants of prostate adenocarcinoma. Although considered rare subtypes of prostate cancer, intraductal/ductal morphological features were observed in 48% of specimens that harbored a HR DNA repair mutation (vs. 12% of non-mutated patients). Therefore, we speculate that patients with intraductal/ductal features on pathology may harbor a higher prevalence of germline HR DNA repair mutations, although this finding must be confirmed by others. Using a pathology-based screening tool may increase the utility of germline genetic testing in this population and may further point to a patient subset that could be enriched for germline HR gene alterations.

Based on that study, our group plans to implement and prioritize germline genetic screening of patients with recurrent or metastatic prostate cancer who have intraductal/ductal features on pathology review. This approach will allow us to estimate the true prevalence of germline DNA repair lesions in intraductal/ ductal prostate adenocarcinoma in a prospective fashion. Additionally, we are currently conducting a Phase 2 study (the TRIUMPH trial) of the PARP inhibitor rucaparib as a monotherapy in men with metastatic hormone-sensitive prostate cancer who harbor a germline mutation in a HR DNA repair gene (NCT03413995). In this trial, rucaparib will be administered in the absence of concurrent ADT, enabling us to determine the independent effect of PARP inhibition in metastatic HR-deficient prostate cancer. We hypothesize that PARP inhibition as a single agent will induce frequent and durable PSA responses (and objective responses in those with measurable disease) in the setting of intact testosterone. As the only trial globally using PARP inhibition in the absence of ADT in patients with germline HR mutations, we would welcome referral of patients to Johns Hopkins for potential participation.

In conclusion, the discovery of HR DNA repair gene mutations in prostate cancer has reinvigorated the study of both germline and somatic prostate cancer genetics. Genomically-selected clinical trials are now needed to determine if targeted therapy can take advantage of these genetic alterations to produce therapeutic gains for our patients.
